# A Suggestive Contrast

**Published:** 1883-06

**Authors:** 


### A SUGGESTIVE CONTRAST.

A spicy little monthly, the *New York Planet*, speaking of the late commencement of Bellevue Hospital College, says, naively: "The graduates who hailed from New York and the neighboring States received the lion's share of applause, doubtless from their friends being present."

That was just the reason, brother *Planet.* The other young men from more distant States, who got only the dog-under-the-table share of the flowers and applause had no sympathizing friends there. It was all right—just what the young strangers expected, doubtless, under the circumstances, and no great harm befell anybody.

It is therefore in no spirit of censure that we refer to the statement of the editor, at all, but only to use it as a tex from which to point a lesson and a moral. We would fain present our New York cotemporary a picture in contrast with the scene at Belleveu, which may surprise him in some degree—may serve to raise him to a higher and more charitable view of human nature on this sin-blasted planet.

We have had since the late war between the States—not a few young men from the North and Northwest—from Main, Vermont, Massachusetts, New York, Michigan, and Ohio, to become matriculants and graduates of the Atlanta and Southern Medical Colleges of this city. Some of these young men carried off the first honors, and many of the most coveted prizes of the colleges, and when they stood upon the stage the night of commencement, surrounded by the beauty and intellect of this representative Southern city, the applaudits and flowers with which they were greeted could not have been more abundant or more heartily given had the seats been crowded with their own kinded and friends from home. Each one, whether a stranger from distant Massachusetts, or from just across the Savannah in South Carolina, received a lion's share alike of applause, if he had won it.

How are these different manifestations to be accounted for? Is it due to an innate difference between the two peoples? A few cynics would answer yea. We do not so think. It is climate makes all the difference. There is an influence in the rays of the Southern sun, in the fragance of Southern flowers, and in the South's exhilerating atmosphere that imparts peculiar energies and manifestations to both intellect and soul.

The moment a stranger from a more northern clime breathes the southern air, he feels the stirings of new soul impulses, if he have a soul. He is conscious of a fellow-feeling—a kindliness towards others and a love for his neighbor that he never felt so deeply and broadly before.

The much derided, so-called chivalry of the Southern people, is nothing more than a unique nobility of character—born of their genial sun; and they are as innocent of that innate selfrespect and elevation of sentiment, the true type of chivalry among any people, as the Switzer's love of his mountain home, or the Laplander's attachment to the snow-mantled plains of his Arctic country.

But the principal object we had before us in writing this article, is to impress the Northern people with the great importance of the South as an educational sanitarium for their youth. No where else on this continent can so many elements be found combined, that are essential to the complete development of women and men.

Our Medical Colleges, as well as literary institutions of every grade, will teem with students from the North, so soon as there is a more general apprehension there of this fact. We offer this as the infallible cure of tuberculosis, phthisis pulmonalis, narrow sectionalism, inordinate negrophilism, and it must gradually impart to both people, a moral, physical and intellectual stamina, which the enlightened world will pronounce the noblest of all the English speaking peoples.

